# Comparison of preservation rhinoplasty versus conventional rhinoplasty techniques: a systematic review of aesthetic and functional outcomes

**DOI:** 10.3389/fsurg.2026.1788643

**Published:** 2026-07-14

**Authors:** Anas Bassam Barnawi, Abdulelah F. Alshehri, Abdulmohsen Alanazi, Faisal Almutairi, Turki Ahmed Aljuhani, Feras Alkholaiwi

**Affiliations:** 1 College of Medicine, Imam Mohammad Ibn Saud Islamic University (IMSIU), Riyadh, Saudi Arabia; 2 Department of Otorhinolaryngology, Head and Neck Surgery, College of Medicine, Imam Mohammad Ibn Saud Islamic University (IMSIU), Riyadh, Saudi Arabia

**Keywords:** aesthetic outcomes, functional outcomes, preservation rhinoplasty, structural rhinoplasty, conventional rhinoplasty, systematic review

## Abstract

**Background:**

Preservation rhinoplasty (PR) has emerged as a technique that prioritizes the conservation of native nasal structures to enhance both functional and aesthetic outcomes. This systematic review compares PR with conventional rhinoplasty (CR), evaluating postoperative satisfaction, nasal airflow, and complications.

**Objective:**

To systematically compare preservation rhinoplasty and conventional rhinoplasty techniques regarding both aesthetic and functional outcomes, by synthesizing evidence from comparative studies that utilized validated outcome measures, and to evaluate the complication rates, patient selection criteria, and methodological variations influencing results.

**Methods:**

Following PRISMA guidelines, we analyzed comparative studies evaluating aesthetic and functional outcomes using validated tools, including Health Nasal Outcomes Survey – Obstruction subscale (SCHNOS-O), Visual Analog Scale – Obstruction (VAS-O), and acoustic rhinometry. Data were extracted from studies across multiple regions and surgical approaches, including the subdorsal strip and spare roof techniques (SRT).

**Results:**

Across five high-quality studies, PR consistently achieved significant enhancements in both SCHNOS-O and VAS-O scores. In Zarei et al., SCHNOS-O improved from 6.65 ± 6.72 to 3.06 ± 4.04 (*p* < 0.05), and VAS-O increased from 7.15 ± 3.31 to 8.56 ± 2.02. These values were statistically higher than pre-op, reflecting improved function. Similarly, CR showed no significant difference in some parameters, though postoperative values still improved. Complication rates were low across both groups; Ferreira et al. reported only minor early complications and similar revision rates between SRT (4/125) and CR (5/125). No major complications occurred in Zarei et al., with only two revision cases after one year. Functional outcomes measured by rhinomanometry and acoustic rhinometry showed no significant difference between PR and CR, indicating preserved nasal patency.

**Conclusion:**

Preservation rhinoplasty may provide comparable safety and functional outcomes to conventional rhinoplasty, with potential aesthetic advantages in carefully selected patients. However, current evidence remains limited by heterogeneity in surgical techniques, outcome measures, and follow-up duration. Further standardized, multicenter, long-term studies are needed to refine patient selection, surgical decision-making, and the broader applicability of preservation techniques.

**Systematic Review Registration:**

https://www.crd.york.ac.uk/prospero/display_record.php?ID=CRD420251090248, PROSPERO: CRD420251090248.

## 1 Introduction

Rhinoplasty is one of the most performed facial plastic surgeries in the world, both for cosmetic and functional purposes. In 2022, over 1.1 million rhinoplasty procedures were performed worldwide, representing a 21.6% rise from the previous year (1). The trend in rhinoplasty techniques is a continued effort towards better postoperative outcomes with fewer complications (2). Rhinoplasty represents one of the most complex facial plastic surgeries, historically defined by two competing philosophies: conventional structural rhinoplasty and preservation rhinoplasty**.** Conventional techniques involve dorsal hump resection followed by reconstruction using spreader grafts or flaps (3). This approach may disrupt the keystone area (the critical junction of nasal bones, upper lateral cartilages, and septum), potentially causing instability or functional compromise (3, 4). In contrast, preservation rhinoplasty (PR) prioritizes minimal tissue disruption, employing push-down or pull-down techniques to reposition the dorsal complex while preserving ligamentous support and vascularity (5, 6). Modern refinements like ultrasonic instrumentation further enhance precision (7). Aesthetically, preservation rhinoplasty yields smoother dorsal contours by avoiding open-roof deformities, resulting in higher early patient satisfaction (<6 months) due to reduced edema (8). However, randomized trials confirm long-term equivalence in dorsal contour, tip symmetry, and patient-rated cosmesis between both techniques at 12–18 months (9, 10). Functionally, while PR theoretically reduces internal valve collapse risks, objective measures show comparable obstruction relief to conventional techniques at 1 year (10, 11). PR's tissue-sparing approach reduces bruising, edema, and recovery time (2–3 weeks vs. 4–6 weeks) (12). Complication profiles differ; conventional techniques carry a 5%–10% risk of midvault collapse (1, 9), while PR may result in 3%–5% residual humps from undercorrection (8). Revision rates remain comparable (2%–4%) for both approaches (8, 11). Patient selection is critical. PR excels in primary cases with moderate humps and thin-to-medium skin (5, 8), while conventional techniques suit severe deviations, thick skin, or >5 mm reductions (1, 9). Surgeon expertise significantly influences outcomes, with hybrid approaches gaining traction for complex anatomies (7, 12). The objective of this study is to systematically compare and evaluate the functional and aesthetic outcomes of preservation rhinoplasty versus conventional rhinoplasty, concerning their relative efficacy, complication rates, and applicability across diverse patient populations. Ultimately, this comparative analysis aims to establish evidence-based best practices and enhance surgical decision-making for personalized, outcomes-oriented rhinoplasty management.

## 2 Materials and methods

### 2.1 Protocol registration

This systematic review followed a predefined protocol registered with PROSPERO (CRD420251090248) (13). Following PRISMA (Preferred Reporting Items for Systematic Reviews) methodology, an exhaustive systematic literature search was conducted (14).

### 2.2 Eligibility criteria

Studies meeting these predefined eligibility requirements were included: (1) direct comparison between preservation rhinoplasty (PR) and conventional rhinoplasty (CR) techniques; (2) inclusion of adult patients aged 18 years or older; (3) a minimum sample size of 10 patients per intervention group; (4) published in peer-reviewed journals; and (5) reporting of at least one quantifiable aesthetic or functional outcome. Studies were excluded if they were non-comparative in design, included pediatric populations, were published in non-English languages, or failed to report relevant quantitative outcome data.

### 2.3 Information sources and search strategy

An exhaustive literature search was performed across PubMed/MEDLINE, Web of Science, and the Cochrane Library, encompassing publications from January 2010 to April 2024. The search methodology incorporated both free-text keywords and controlled Medical Subject Headings (MeSH) terms, including “preservation rhinoplasty,” “dorsal preservation,” “structural rhinoplasty,” “conventional rhinoplasty,” “aesthetic outcomes,” “functional outcomes,” and “complications.” An example PubMed search string was: (“preservation rhinoplasty”[Title/Abstract] OR “dorsal preservation”[Title/Abstract]) AND (“conventional rhinoplasty”[Title/Abstract] OR “structural rhinoplasty”[Title/Abstract]) AND (“outcome”[Title/Abstract] OR “complication”[Title/Abstract] OR “revision”[Title/Abstract]).

### 2.4 Study selection

**Figure 1:**
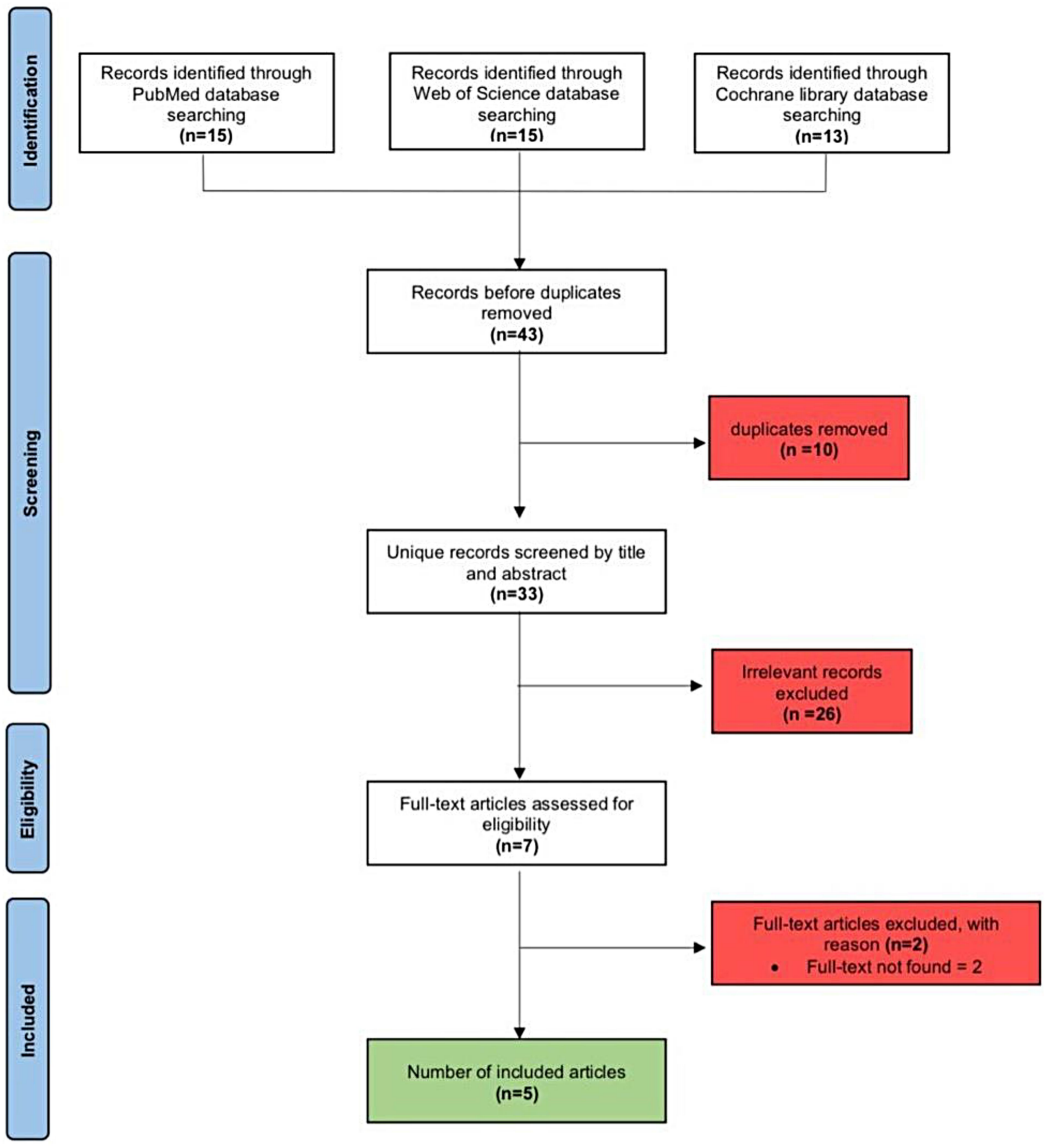
PRISMA flow diagram summarizing the screening process.

Following the removal of duplicates, all identified records were systematically imported into Rayyan, a web-based systematic review management platform (15). Two reviewers independently evaluated titles and abstracts for potential inclusion. Full texts of relevant studies were subsequently reviewed against eligibility criteria. Discrepancies were resolved through discussion or, when necessary, by arbitration with a third reviewer. The selection protocol followed PRISMA 2020 standards. The complete study selection process is summarized in Figure 1.

### 2.5 Data extraction

Data were extracted independently by four reviewers utilizing a standardized extraction form developed in Microsoft Excel. Extracted variables included study characteristics (e.g., author, publication year, country, and study design), patient demographics, surgical techniques employed, duration of follow-up, reported outcome measures (e.g., NOSE, SCHNOS, VAS), and postoperative complications (e.g., revision surgery, residual deformities). In instances of missing or unclear data, attempts were made to contact the corresponding authors.

### 2.6 Risk of bias assessment

Methodological quality assessment was performed using validated tools appropriate to each study design. Randomized controlled trials (RCTs) were evaluated with the Cochrane Risk of Bias 2 (ROB-2) tool. At the same time, observational studies were assessed using the Newcastle-Ottawa Scale (NOS), which examines three critical domains: (1) selection of study groups, (2) comparability of groups, and (3) ascertainment of outcomes. To ensure reliability, two independent reviewers conducted all assessments, with any discrepancies resolved through consensus discussion.

### 2.7 Outcome measures and synthesis strategy

Continuous outcome variables, such as SCHNOS and VAS scores, were extracted as means and standard deviations, while categorical outcomes, including complication and revision rates, were expressed as proportions. Due to heterogeneity in outcome definitions, measurement tools, and follow-up intervals, a meta-analysis was not performed. Instead, a descriptive synthesis of findings was undertaken, emphasizing the directionality and consistency of reported results across included studies.

### 2.8 Quality assessment and risk of bias

**Figure 2:**
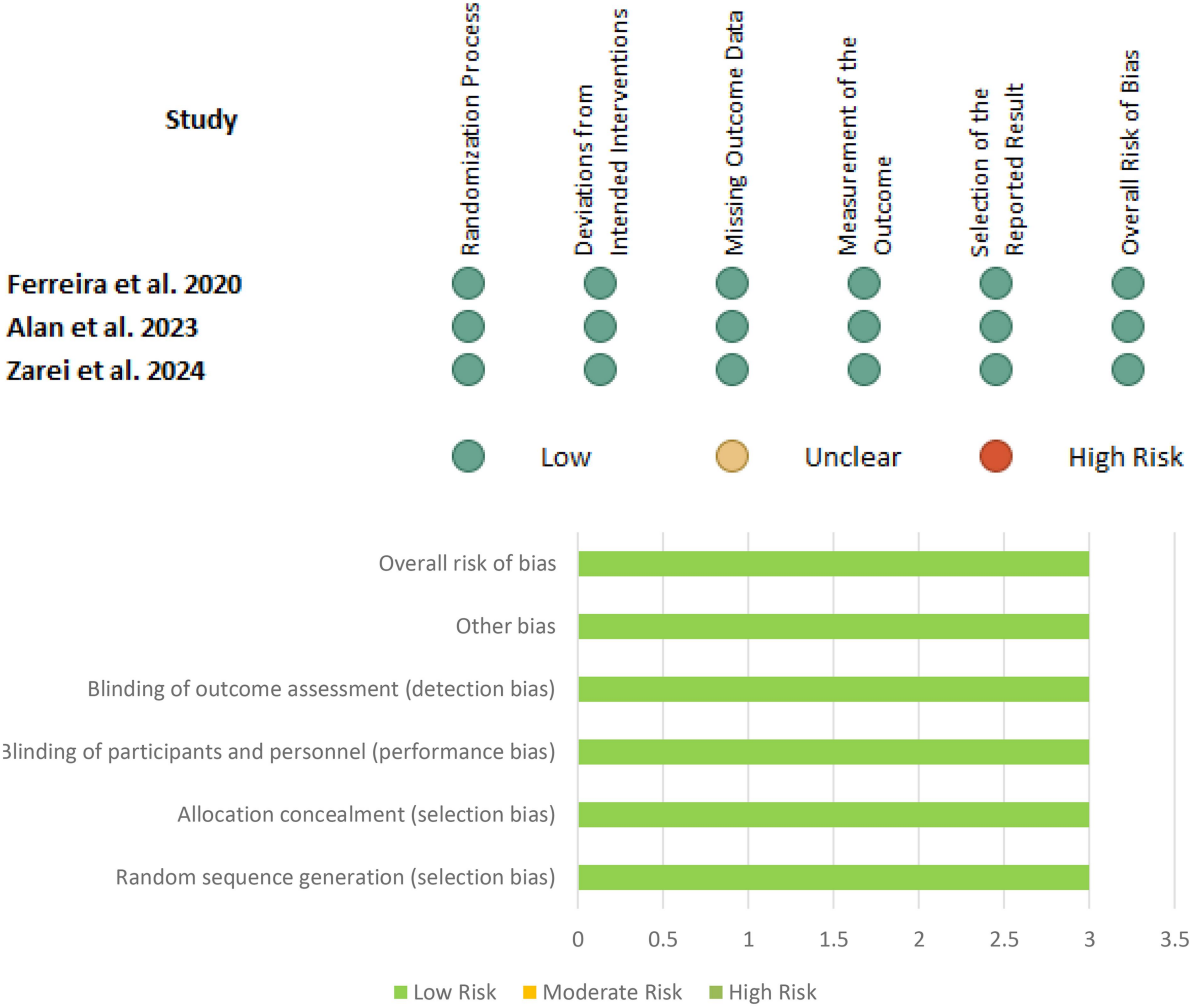
Risk of bias assessment of the included RCTs using cochrane ROB-2.

The risk of bias for cohort studies included was assessed with the Newcastle-Ottawa Scale (NOS) (20). The NOS rates studies on articles based on three categories: Selection (maximum 4 stars), Comparability (maximum 2 stars), and Outcome (maximum 3 stars). Both studies attained the maximum score for the Selection domain (4/4). This is due to uniform selection criteria among studies under inclusion. In the Comparability domain, (16), did not provide evidence of adequate control for confounding variables (16). In the Outcome category, both studies were awarded two of three possible stars because the adequacy of follow-up could not be reported (Table 1). For the RCTs, the risk of bias was assessed using the Cochrane ROB-2 tool (21). All three RCTs, Ferreira et al. 2020, Alan et al. 2023, and Zarei et al. 2024, had low risk of bias across all the domains (9, 18, 19). The assessed domains encompassed the randomization procedure, deviations from intended interventions, incomplete outcome data, outcome measurement methods, and selective reporting of results. (Figure 2).

**Table 1:** Risk of bias assessment of the included cohort studies using NOS.

	Selection				Comparability	Outcome		
Study	Case Def. Adequate?	Representativeness?	Selection of Comparison Group?	Definition of Comparison Group?	Comparability of Groups?	Outcome Ascertainment?	Follow-up: Long Enough?	Adequacy of Follow-up?
Alford et al. (16)	Yes	Yes	Yes	Yes	No	Yes	Yes	(Not reported)
Emre et al. (17)	Yes	Yes	Yes	Yes	Yes	Yes	Yes	(Not reported)

Study	Selection Score	Comparability Score	Outcome Score	Total Score	Risk of Bias Summary
Alford et al. (16)	⋆⋆⋆⋆	0	⋆⋆	6	Moderate
Emre et al. (17)	⋆⋆⋆⋆	⋆⋆	⋆⋆	8	Low

The symbol ⋆ indicates one star/point awarded in the Newcastle-Ottawa Scale (NOS) risk-of-bias assessment.

### 2.9 Characteristics of the included studies

Five studies between the years 2020–2024 were included that had a total of 556 patients from the USA (16), Turkey (17, 19), Portugal (18), and Iran (9). The study designs included three randomized controlled trials, one prospective cohort study, and one retrospective cohort study. Sample sizes were between 34 and 250 participants, with mean ages ranging from 23.0 ± 4.2 to approximately 39 years. Female patients predominated in all studies, comprising 52.9% to 88.6% of participants. All studies compared dorsal preservation (DP) techniques—such as Let-Down, Push-Down, or altered subdorsal strip—versus various structural rhinoplasty techniques, such as classic structural rhinoplasty (CSR), component dorsal reduction (CDR), and standard hump resection (Table 2).

**Table 2:** Characteristics of the included studies.

Study ID	Country	Study Design	Sample Size	Male to Female ratio	Age (Mean ± SD)	Surgical Technique Comparison	Follow-up Duration
Alford et al. (16)	USA	Retrospective Cohort	64	DP: 36.7%/63.3%; NDP: 47.1%/52.9%	∼39 years	Dorsal Preservation (DP) vs. Non-Dorsal Preservation (NDP)	∼14 months
Emre et al. (17)	Turkey	Prospective Cohort	124	DP: 37%/64%; CSR: 47%/53%	25 ± 7.75	Dorsal Preservation (Let-Down/Push-Down) vs. Classical Structural Rhinoplasty (CSR)	6 months
Ferreira et al. (2020)	Portugal	RCT	250	CDR: 35.2%/64.8%; SRT: 32.0%/68.0%	35.2 ± 10.0	Spare Roof Technique (SRT) vs. Component Dorsal Reduction (CDR)	20 months
Alan et al. (19)	Turkey	RCT	34	SR: 52.6%/47.4%; PR: 46.7%/53.3%	23.0 ± 4.2	Preservation Rhinoplasty (PR) vs. Structural Rhinoplasty (SR)	12 months
Zarei et al. (9)	Iran	RCT	84	CHR: 22.0%/78.0%; DPR: 11.4%/88.6%	30.96 ± 6.75	Dorsal Preservation Rhinoplasty (DPR), Modified Subdorsal Strip vs. Conventional Hump Resection (CHR)	∼14 months

RCT, randomized controlled trial; DP, dorsal preservation; NDP, non-dorsal preservation (structural rhinoplasty); CSR, classical structural rhinoplasty; CDR, component dorsal reduction; SRT, spare roof technique; SR, structural rhinoplasty; PR, preservation rhinoplasty; CHR, conventional hump resection; DPR, dorsal preservation rhinoplasty.

## 3 Results

### 3.1 Aesthetic outcomes

The included studies assessed aesthetic outcomes using various methods, including crowdsourced evaluations, patient-reported outcome measures (PROMs) like the Standardized Cosmesis and Health Nasal Outcomes Survey (SCHNOS-C) and visual analog scales (VAS-C), and objective image analysis. Alford et al. 2024 used a crowdsourcing platform to evaluate aesthetic outcomes (16). They found that both dorsal preservation and structural rhinoplasty cohorts were associated with improved overall crowdsourced aesthetic outcomes and improved outcomes across all sub parameters. The delta (improvement from preoperative to postoperative) was 0.300 (95% CI +/- 0.047) for the dorsal preservation cohort and 0.377 (95% CI +/- 0.055) for the structural cohort. The raters demonstrated poor interobserver reliability in distinguishing patients who underwent dorsal preservation rhinoplasty (correlation coefficient: −0.0554; 95% CI ±0.058), indicating that the technique may yield aesthetic outcomes that closely resemble natural, unoperated nasal morphology. In contrast, raters were likely correct in identifying structural rhinoplasty (correlation coefficient 0.74, 95% CI +/- 0.47).

Ferreira et al. (2020) performed a prospective randomized study comparing the Spare Roof Technique (SRT, a preservation approach) with Component Dorsal Hump Reduction (CDR, a structural technique) (18). Nasal aesthetics were assessed using the Utrecht Questionnaire (UQ), incorporating a Visual Analog Scale (VAS) for nasal appearance (0 = very unattractive, 10 = very attractive). Both surgical groups demonstrated statistically significant enhancements in aesthetic VAS scores from baseline to postoperative follow-ups (3 and 12 months). In the Component Dorsal Reduction group (CDRg), mean scores increased from 3.66 [1.36] preoperatively to 7.00 [2.04] at 3 months and 7.35 [2.13] at 12 months. Similarly, the Spare Roof Technique group (SRTg) improved from 3.81 [1.29] preoperatively to 8.14 [1.18] at 3 months and 8.45 [1.10] at 12 months. Notably, comparative analyses revealed significantly greater aesthetic improvement in the SRTg compared to the CDRg at both 3 months (*P* < 0.001) and 12 months (*P* < 0.001) postoperatively. Multivariable analysis further established that surgical technique significantly influenced aesthetic outcomes (SRT demonstrated superior results, *P* < 0.001, with effect sizes of 0.111 at 3 months and 0.107 at 12 months). Additionally, dorsal camouflage type affected outcomes, with Powder Cartilage + Platelet-Rich Plasma (PC + PRP) yielding better aesthetic evaluations than Diced Cartilage (DC) at both 3 months (*P* = 0.004, effect size 0.033) and 12 months (*P* < 0.001, effect size 0.032). Enhanced nasal aesthetic VAS scores were associated with improved subjective body image perception regarding nasal appearance. Both groups also exhibited highly significant improvement in aesthetic Likert-scale assessments following rhinoplasty (*P* < 0.001).

Alan et al. (2023) conducted a comparison between dorsal preservation rhinoplasty (PR) and structural rhinoplasty (SR), assessing aesthetic outcomes using the SCHNOS-C questionnaire at 3 and 12 months postoperatively (19). Their findings revealed significant improvement in SCHNOS-C scores (reflecting enhanced aesthetic results) at both follow-up intervals compared to preoperative baselines in both the SR and PR groups (*P* < 0.001 for SR, *P* = 0.001 for PR). Nevertheless, the study concluded that no statistically significant difference existed between the two groups in patient-reported outcome measures (PROMs), including SCHNOS-C.

Zarei et al. (2024) performed a prospective randomized trial assessing dorsal preservation rhinoplasty (DPR) versus conventional hump resection (CHR) with spreader flap reconstruction (9). Aesthetic outcomes were analyzed after one year using the SCHNOS-C questionnaire, VAS-C scale, and objective image-based measurements (residual hump, nasal width, projection, and rotation). Both surgical groups demonstrated significant postoperative improvement in cosmetic outcomes based on SCHNOS-C and VAS-C scores. In the CHR group, mean SCHNOS-C decreased from 15.53 ± 5.19 preoperatively to 4.00 ± 3.32 postoperatively, while VAS-C improved from 3.22 ± 2.32 to 8.39 ± 1.17. The DPR group showed similar improvement, with SCHNOS-C decreasing from 14.68 ± 4.86 to 3.56 ± 3.25 and VAS-C increasing from 3.74 ± 2.03 to 8.32 ± 1.27. Objective measurements revealed significant reductions in hump size, projection, and width, along with increased tip rotation in both groups. The key finding was that no statistically significant differences existed between DPR and CHR in any postoperative aesthetic outcome measure at one year. Comparative analysis yielded the following *P*-values: SCHNOS-C (*P* = 0.47), VAS-C (*P* = 0.81), residual hump (*P* = 0.26), nasal width (*P* = 0.37), projection (*P* = 0.70), and rotation (*P* = 0.79). Residual hump measurements averaged 0.05 ± 0.14 mm for DPR versus 0.02 ± 0.09 mm for CHR (Table 3).

**Table 3:** Aesthetic outcomes.

Study	Technique (Cohort)	Measurement Method	Preoperative Value	Postoperative Value (3 Months)	Postoperative Value (12 Months)
Alford et al. (16)	Dorsal Preservation	Crowdsourced Evaluation	Not applicable	Improvement: 0.300 (95% CI +/- 0.047)	Not applicable
	Structural Rhinoplasty	Crowdsourced Evaluation	Not applicable	Improvement: 0.377 (95% CI +/- 0.055)	Not applicable
Ferreira et al. (18)	Component Dorsal Hump Reduction (CDR)	VAS for Nasal Appearance (0–10)	Mean: 3.66 [1.36]	Mean: 7.00 [2.04]	Mean: 7.35 [2.13]
	Spare Roof Technique (SRT)	VAS for Nasal Appearance (0–10)	Mean: 3.81 [1.29]	Mean: 8.14 [1.18]	Mean: 8.45 [1.10]
Alan et al. (19)	Structural Rhinoplasty (SR)	SCHNOS-C	Not specified	Significant improvement (*P* < 0.001)	Significant improvement (*P* < 0.001)
	Preservation Rhinoplasty (PR)	SCHNOS-C	Not specified	Significant improvement (*P* = 0.001)	Significant improvement (*P* = 0.001)
Zarei et al. (9)	Conventional Hump Resection (CHR)	SCHNOS-C	15.53 ± 5.19	Not applicable	4.00 ± 3.32
		VAS-C	3.22 ± 2.32	Not applicable	8.39 ± 1.17
	Dorsal Preservation Rhinoplasty (DPR)	SCHNOS-C	14.68 ± 4.86	Not applicable	3.56 ± 3.25
		VAS-C	3.74 ± 2.03	Not applicable	8.32 ± 1.27
		Residual Hump	Not specified	Not applicable	CHR: 0.02 ± 0.09 mm; DPR: 0.05 ± 0.14 mm

### 3.2 Functional outcomes

Functional outcomes were assessed using PROMs like the Nasal Obstruction Symptom Evaluation (NOSE) and Standardized Cosmesis and SCHNOS-O, and objective measures like acoustic rhinometry [Minimum Cross-Sectional Area, Nasal Cavity Volume (MCA, VOL)] or rhinomanometry [Total Nasal Volume, Total Nasal Resistance (TNV, TNR)]. Emre et al. 2024 primarily focused on comparing nasal airway dimensions after Classical Structural Rhinoplasty (CSR) and Dorsal Preservation Rhinoplasty (DPR) using acoustic rhinometry (AR) (17). They measured minimum cross-sectional areas (MCA) and internal nasal volumes (VOL). Patients in both DPR and CSR groups had similar nasal airway dimensions before and after surgery. There was no statistically significant difference between preoperative and postoperative MCA and VOL levels in either DPR or CSR technique, and no significant differences between the 2 techniques regarding VOL and MCA. For example, MCA1 left side DPR vs CSR *P* = 0.539 preoperatively and *P* = 0.905 postoperatively; VOL1 right side DPR vs. CSR *P* = 0.843 preoperatively and *P* = 0.114 postoperatively. They also used the NOSE questionnaire and reported that all patients had a NOSE score less than 5 pre- and post-surgery, indicating no negative subjective functional outcomes. Their results suggest that the technique (DPR vs. CSR) is not significantly related to any changes in nasal airway dimensions and does not significantly affect geometry or ease of breathing.

Ferreira et al. 2020 evaluated functional outcomes using a VAS score for nasal patency (0 = very bad, 10 = very good) for each side (18). Analyses showed a significant and consistent improvement in functional VAS scores on both sides in both groups. Postoperative mean functional VAS scores were: Right side CDRg 7.46 [1.51] at 3 months and 7.43 [1.57] at 12 months; Right side SRTg 8.13 [1.17] at 3 months and 8.10 [1.57] at 12 months. Left side CDRg 8.02 [1.18] at 3 months and 8.11 [1.29] at 12 months; Left side SRTg 8.02 [1.35] at 3 months and 8.69 [1.32] at 12 months. The SRTg demonstrated superior functional outcomes compared to the CDRg at 3 months postoperatively (right side: *P* < 0.001; left side: *P* = 0.960). By 12 months, the SRTg showed statistically significant improvement bilaterally (*P* = 0.001). These enhanced functional results in the SRTg were partially explained by anatomical changes, particularly widening of the middle nasal vault and internal nasal valve area.

Alan et al. (2023) assessed functional outcomes through the use of NOSE, SCHNOS-O, and rhinomanometry (TNV, TNR) (19). They reported that NOSE and SCHNOS-O scores showed significant improvement at the 3rd and 12th postoperative months compared to the preoperative period in both groups. Nevertheless, no significant differences were observed between the groups regarding PROMs (NOSE, SCHNOS-O). Rhinomanometric evaluation revealed no significant intergroup differences in TNV or TNR. The mean total nasal volume (TNV) at the 12th month was significantly higher than the preoperative measurement in the PR group (*P* = 0.031), whereas no such change was noted in the SR group, and no significant difference was found between the groups concerning TNV or TNR. The authors also observed that their rhinomanometric findings did not correlate with the PROMs in their study.

Zarei et al. 2024 evaluated functional outcomes using SCHNOS-O and VAS-O after 1 year (9). Patients in both groups reported significantly improved functional outcomes based on these scales after surgery. For CHR, pre-op SCHNOS-O was 4.73 ± 6.66, post-op was 2.63 ± 4.54; pre-op VAS-O was 7.92 ± 2.98, post-op was 8.80 ± 1.83. For DPR, pre-op SCHNOS-O was 6.65 ± 6.72, post-op was 3.06 ± 4.04; pre-op VAS-O was 7.15 ± 3.31, post-op was 8.56 ± 2.02. They found no significant differences in postoperative functional outcomes (SCHNOS-O, VAS-O) between the dorsal preservation group and the conventional hump resection group after 1 year (Table 4).

**Table 4:** Functional outcomes.

Study	Technique (Cohort)	Measurement (Side)	Pre-op	3 Months	12 Months	(Pre- vs. Post-op)	*P*-value (Inter-group)
Emre et al. (17)	DPR (left)	MCA1	-	-	-	-	Pre-op: 0.539; Post-op: 0.905
	CSR (left)	MCA1	-	-	-	-	
	DPR (right)	VOL1	-	-	-	-	Pre-op: 0.843; Post-op: 0.114
	CSR (right)	VOL1	-	-	-	-	
	All Patients	NOSE score	<5	<5	-	-	-
Ferreira et al. (18)	CDRg (Right)	VAS Patency	-	7.46 [1.51]	7.43 [1.57]	Significant Improvement	3 Months: <0.001
	SRTg (Right)	VAS Patency	-	8.13 [1.17]	8.10 [1.57]	Significant Improvement	12 Months: 0.001
	CDRg (Left)	VAS Patency	-	8.02 [1.18]	8.11 [1.29]	Significant Improvement	3 Months: 0.960
	SRTg (Left)	VAS Patency	-	8.02 [1.35]	8.69 [1.32]	Significant Improvement	12 Months: 0.001
Alan et al. (19)	PR	TNV	-	-	Statistically higher than pre-op	Significant Improvement	No significant difference
	SR	TNV	-	-	Not statistically higher than pre-op	-	No significant difference
Zarei et al. (9)	CHR	SCHNOS-O	4.73 ± 6.66	-	2.63 ± 4.54	Significant Improvement	0.466
		VAS-O	7.92 ± 2.98	-	8.80 ± 1.83	Significant Improvement	0.362
	DPR	SCHNOS-O	6.65 ± 6.72	-	3.06 ± 4.04	Significant Improvement	
		VAS-O	7.15 ± 3.31	-	8.56 ± 2.02	Significant Improvement	

DPR, dorsal preservation rhinoplasty; CSR, classical structural rhinoplasty; MCA1, minimum cross-sectional area 1; VOL1, volume 1; NOSE, nasal obstruction symptom evaluation; VAS, visual analog scale; CDRg, component dorsal reduction group; SRTg, spare roof technique group; TNV, total nasal volume; TNR, total nasal resistance; PR, preservation rhinoplasty; SR, structural rhinoplasty; SCHNOS-O, standardized cosmesis and health nasal outcomes survey – obstruction; VAS-0, visual analog scale – obstruction.

### 3.3 Complications

The sources provide limited details on complications, focusing primarily on outcomes related to the dorsal modification. Ferreira et al. (2020) documented three instances of complications in a cohort of 250 patients: epistaxis (occurring within 48 h postoperatively, one case in the SRTg group) and tip infection (one case in the CDRg group and one in the SRTg group) (18). Over the follow-up duration (averaging 20 months), nine patients underwent revision surgery due to aesthetic concerns related to the dorsum (five cases in the CDRg group and four in the SRTg group). The primary indications for revision included residual bony hump (two cases) and cartilaginous hump (two cases) in the SRTg group, as well as dorsal irregularities in the CDRg group. Zarei et al. (2024) reported no significant complications in either group within their study of 84 patients after a one-year period (9). A single patient in the DPR group necessitated a radix graft, while two patients (one from each group) required revision surgery after one year due to dorsal irregularity in the DPR patient and tip asymmetry in the CHR patient.

### 3.4 Other outcomes and methodologies of the included studies

Other outcomes reported include assessment methods, factors influencing outcomes, and overall comparability. Alford et al. 2024 highlight the use of a crowdsourcing platform as a valuable tool for measuring aesthetic outcomes in plastic surgery (16). This method allowed for a more objective analysis of the inherent subjectivity in aesthetic evaluations.

In addition, Ferreira et al. 2020 used the Utrecht Questionnaire (UQ) and VAS scales, noting that PROMs are equally important as photographic documentation (18). Their multivariable analysis identified surgical technique (SRT better) and type of dorsal camouflage (PC + PRP better) as factors influencing aesthetic evaluation. They found no significant correlation between the initial hump size (even >5 mm) and final aesthetic outcomes for either technique. They noted that results were slightly better at 12 months than at 3 months post-surgery in both groups, suggesting continued improvement. The revisional surgery rate was similar between groups (4/125 SRTg vs. 5/125 CDRg). On the other hand, Alan et al. 2023 utilized PROMs (NOSE, SCHNOS) and rhinomanometry, noting their study was the first to compare these techniques using rhinomanometry (19). They found good functional and aesthetic results with both techniques. Zarei et al. 2024 used SCHNOS, VAS, and objective image analysis (9). They noted that selection criteria (septal cartilage amount, rhinion position, hump shape, bony pyramid width/deviation, septal deviation) must be considered for DPR candidates. However, their study presented certain limitations, such as a constrained sample size and specific inclusion criteria, which may limit the generalizability of the findings to cases involving larger humps or deviated noses. They concluded that the modified subdorsal strip technique for dorsal preservation rhinoplasty (DPR) may yield aesthetic and functional outcomes comparable to those of conventional hump resection with spreader flap reconstruction.

## 4 Discussion

This systematic review represents a comprehensive and critical comparison between preservation rhinoplasty (PR) and conventional rhinoplasty (CR), assessing both aesthetic and functional outcomes. The review consolidates findings from five high-quality comparative investigations, encompassing randomized controlled trials and prospective cohort studies, which were carried out among varied demographic groups. Collectively, the evidence supports the growing prominence of preservation techniques in modern rhinoplasty practice.

Aesthetic outcomes in the included studies consistently favored PR techniques, particularly the Spare Roof Technique (SRT), subdorsal strip, and push-down approaches. Ferreira et al. demonstrated significantly superior aesthetic improvement in the PR group using validated tools such as the Utrecht Questionnaire and VAS scores, with results sustained at 12 months postoperatively (18). Similarly, Alan et al. and Zarei et al. found no significant differences in postoperative aesthetic scores between PR and CR groups, though both reported substantial improvements in VAS-C and SCHNOS-C values across time points (9, 19). The ability of PR to preserve the native dorsum contour appears to contribute to a more “unoperated” aesthetic result—an important consideration in facial harmony.

Functional outcomes were similarly favorable (17). used acoustic rhinometry to compare pre- and postoperative nasal airflow parameters (MCA, VOL), and no statistically significant differences were observed between the PR and CR groups. indicating functional stability in both approaches (17). Alan et al. reinforced these findings using both subjective (NOSE, SCHNOS-O) and objective (rhinomanometry) metrics, confirming that neither technique compromised nasal patency (19). Notably, Ferreira et al. observed significantly higher functional VAS scores in the PR cohort at 12 months, possibly due to better preservation of the internal nasal valve (18). These findings challenge the traditional belief that structural techniques are inherently superior in maintaining nasal function.

Our findings highlight several key strengths of this review. First, the review was conducted in adherence to PRISMA guidelines, with strict inclusion criteria requiring comparative design and quantitative outcome reporting. Second, a wide range of validated measurement tools—including PROMs (e.g., NOSE, SCHNOS, VAS) and objective tests (e.g., rhinomanometry, acoustic rhinometry)—were included to provide a multidimensional understanding of outcomes. Third, the review incorporated international studies, thus offering insight into anatomical variability and technique generalizability across ethnic groups.

Despite these strengths, the review is not without limitations. There was methodological heterogeneity across studies, including variability in the surgical techniques classified under PR and CR. For instance, the SRT used by Ferreira et al. differs technically from the modified subdorsal strip approach used by Zarei et al. (9, 18). This distinction is important because preservation rhinoplasty represents an umbrella term rather than a single standardized operation. Contemporary preservation approaches may include dorsal preservation techniques, subdorsal or low-strip modifications, hybrid structural-preservation rhinoplasty, and ligament-, cartilage-, or soft-tissue-preservation concepts (22–24). Therefore, techniques such as the Spare Roof Technique, subdorsal strip methods, structural preservation rhinoplasty, and endonasal ligament-preservation rhinoplasty should not be interpreted as interchangeable. This technique-level heterogeneity limits direct comparison across studies and requires cautious interpretation of the overall findings. Follow-up durations also varied, with only one study providing data beyond 12 months. Furthermore, many studies lacked stratification by critical variables such as skin thickness, dorsal hump size, and surgeon experience—factors known to influence outcomes. Additionally, while PROMs are valuable tools, their subjective nature may introduce bias, particularly in open-label surgical trials.

Clinically, the findings of this review support the judicious use of preservation rhinoplasty in primary cases with favorable anatomy, particularly patients with straight nasal profiles, moderate dorsal humps, and thin to medium skin. PR offers advantages such as reduced operative trauma, faster recovery, and more natural dorsal lines. However, in patients with complex deformities (e.g., S-shaped humps, significant septal deviation, thick skin), conventional structural rhinoplasty or hybrid approaches may be more appropriate. Zarei et al. highlighted that while PR and CR yielded similar objective results, revision was required in cases with residual bony hump, underscoring the importance of case selection (9).

Finally, this review emphasizes the need for future research to address gaps in current evidence. Future studies should emphasize clearer documentation and classification of surgical techniques to improve reproducibility, while preserving flexibility for patient-specific customization. Multicenter randomized controlled trials with uniform PROMs and objective outcome tools are necessary to establish definitive practice guidelines. Additionally, the integration of 3D imaging and computational modeling may further refine preoperative planning and postoperative evaluation.

## 5 Conclusion

Evidence from this review indicates that preservation rhinoplasty offers comparable safety and functional outcomes to conventional approaches, with potential aesthetic benefits in well-selected cases. Across multiple high-quality studies, PR consistently maintained or improved functional outcomes and achieved high patient satisfaction, often rivaling or exceeding CR in aesthetic measures. While limitations exist, including heterogeneity in surgical technique and short follow-up periods in some studies, the overall evidence supports wider adoption of preservation methods. Surgeons should continue to refine patient selection criteria and integrate objective tools into preoperative planning. Ultimately, preservation rhinoplasty reflects a paradigm shift in modern nasal surgery—from reconstruction to conservation—aligning technical innovation with functional and aesthetic integrity.

## 6 Protocol registration

The review protocol was registered in PROSPERO under the registration number CRD420251090248.

## Data Availability

The original contributions presented in the study are included in the article/Supplementary Material, further inquiries can be directed to the corresponding author.
